# Exercise Preferences in Young Adults with Cancer—The YOUEX Study

**DOI:** 10.3390/curroncol30020113

**Published:** 2023-01-21

**Authors:** Annelie Voland, Verena Krell, Miriam Götte, Timo Niels, Maximilian Köppel, Joachim Wiskemann

**Affiliations:** 1 Department of Medical Oncology, National Center for Tumor Diseases, Heidelberg University Hospital, 69120 Heidelberg, Germany; 2 Department of Sports Medicine, Charité—Universitätsmedizin Berlin, 10115 Berlin, Germany; 3 Department of Sports Medicine, Humboldt—Universität zu Berlin, 10115 Berlin, Germany; 4 West German Cancer Center, University Hospital Essen, 45122 Essen, Germany; 5 Department I of Internal Medicine, Center of Integrated Oncology Aachen Bonn Cologne Düsseldorf, University Hospital of Cologne, 50937 Cologne, Germany

**Keywords:** exercise, oncology, adolescents and young adults (AYA), breast cancer, physical activity, online exercise programs, COVID-19

## Abstract

(1) Background: Strong evidence supports the persuasive positive effects of exercise for cancer patients and survivors. Different approaches of exercise programs have been established; however, the special interests of young adults (YAs) with cancer have rarely been considered in exercise interventions. Therefore, the study YOUng EXercisers (YOUEX) aimed to investigate exercise preferences in YAs. (2) Methods: YOUEX was a three-arm, patient preference-based non-randomized, longitudinal, pre–post exercise intervention, offering three different exercise modules to YAs during or after acute therapy (Module 1: online supervised group-based (M1); Module 2: online unsupervised (M2); Module 3: in-person supervised (M3)). The intervention period was 12 weeks with another 12-week follow-up period, the modules could be changed or amended after 6 and 12 weeks. (3) Results: 92 YAs were allocated to the study. At baseline, 50 YAs (54%) chose M2, 32 YAs (35%) M1 and 10 YAs (11%) M3. The analysis revealed high acceptability and feasibility of the online exercise programs (M1, M2). There was a high impact of the COVID-19 pandemic on the execution of M3. YAs showed diverse preferences in module selection due to differences in, e.g., cancer therapy status or favored level of supervision. (4) Conclusions: YAs need personalized exercise programs that consider their individual interests and needs. Online exercise programs can be a promising addition to existing exercise opportunities. They are an effective way to increase physical activity levels in YAs.

## 1. Introduction

A strong body of evidence demonstrates the beneficial psychological and physiological effects of physical activity (PA) and exercise in cancer patients and survivors before, during and after treatment. Hundreds of exercise interventions have revealed the reduction in highly prevalent cancer- and treatment-related side effects, such as fatigue [1,2], physical disabilities [3,4], polyneuropathy [5,6,7], or lymphedema [8,9]. Several systematic reviews and meta-analyses have shown the positive effects on overall quality of life [10,11,12]. Moreover, regular PA during and after cancer treatment is associated with improved treatment efficacy [13] and increased cancer-specific survival rates [14]. Based on the high amount of evidence, the American College of Sports Medicine (ACSM) defined specific exercise guidelines for individual side effects in oncology. They recommend to reduce sedentary time [15] and to reach at least 150 min of moderate-intensity exercise (or 75 min of vigorous-intensity exercise) and two strength-training sessions per week [16]. These recommendations correspond to the World Health Organization guidelines on physical activity and sedentary behavior [17]. However, to date, the vast majority of studies have been conducted with cancer patients over the age of 50 years, underrepresenting young adults and their special needs and preferences [18,19]. A review by Munsie et al. [20] highlights the lack of high-quality studies that examine the effects of physical activity in this cohort.

Commonly, the term ‘adolescents and young adults’ (AYA) includes individuals between the ages of 15–39 years. Today, cancer occurs about 66,000 times in AYA per year in Europe [21]. Due to improvements in treatment and care, the five-year relative survival of AYA diagnosed with cancer is 80–85%. However, long treatment regimens and periods of isolation away from their peer groups compromise their physical and psychological well-being. Further, long-term sequelae of cancer treatments can range from mild to severe. Late effects involve, for example, cardiovascular diseases, lung problems, high risk for osteoporosis or increased risks to develop other types of cancer later in life [18,22]. In light of the special life situation of AYA, the adoption and implementation of exercise programs need to involve adjustments according to the various factors, such as physical and mental health, financial position, time, and family role [23]. At present, there are very few specialized exercise programs that focus on the interests and needs of AYA. Most of them are tailored for children undergoing cancer treatment or childhood cancer survivors [24,25,26,27,28,29]. According to reports of the German Foundation for Young Adults with Cancer, there is a lack of attractive exercise programs for young adults with cancer aged 18–39 (YAs) as well as a lack of research about their feasibility and efficacy [30]. Although most YAs are highly interested in PA support and increasing PA levels [31], only a few studies have examined the feasibility and acceptance of exercise programs in YA [19].

With a focus on YAs, we developed a health care research study, called YOUEX (YOUng EXercisers) that addresses and investigates the needs and preferences of YAs aged 18–39 years to participate in a structured exercise program. Therefore, we implemented three different exercise modules that included different online tools or in-person training sessions. The YOUEX study is based on a comprehensive evaluation design and is supported by the German Foundation for Young Adults with Cancer.

The goal of the YOUEX study was to investigate the feasibility, acceptance and individual module selection of the three exercise modules by YAs with cancer to gain knowledge about how exercise programs should be structured for this young target group.

## 2. Materials and Methods

### 2.1. Study Design

We conducted a three-arm, patient preference-based non-randomized longitudinal pre-post exercise intervention for YAs with cancer with three eligible exercise modules. The main intervention period was 12 weeks with another 12-week follow-up period. We defined four time points for the intervention evaluation (T0: baseline; T1: after 6 weeks of intervention; T2: 12 weeks of intervention; T3: follow-up 24 weeks). The study protocol was approved by the ethics committee of the medical faculty at Heidelberg University (S-932/2020). The study was registered at clinicaltrials.gov (NCT05613699).

### 2.2. Participants and Recruitment

Participants were eligible if they were aged between 18–39 years, had a cancer diagnosis within the past five years and confirmed the study letter of consent. Exclusion criteria were the lack of physical exercise clearance from the attending oncologist (e.g., in case of fragile bone metastases), subjectively perceived cancer-related cognitive impairment, current participation in another exercise intervention or insufficient German language skills. We recruited patients via social media, clinical websites, flyers and from survivor groups of the German Foundation for Young Adults with Cancer from September 2020 to April 2021. Interested patients were contacted via e-mail or telephone for further information and to check inclusion criteria.

### 2.3. YOUEX Exercise Intervention Modules

Patients who fulfilled the inclusion criteria and provided informed consent received a comprehensive exercise consultation via phone or in person at the exercise department of the National Center of Tumor Disease (NCT), Heidelberg, or the department of sports medicine at Charité—Universitätsmedizin, Berlin. In the first consultation, study coordinators collected information about the cancer diagnosis and therapy, cancer- and cancer-treatment-related side effects, medical history, past and current physical activity levels and patient’s preferences to exercise. Further, they explained to the participants that they were free to choose one of three different exercise programs. The different modules were developed in exchange with the German Foundation for Young Adults with Cancer. Based on a survey that was carried out by the foundation and asked young adults for their wishes regarding different exercise options, the following three modules were developed:

1. Module 1 (M1): supervised, group-based, online exercise program once a week
2. Module 2 (M2): unsupervised, individual home-based training with an online-training app at least once per week
3. Module 3 (M3): participation in a supervised, in-person exercise program close to place of residence at least once per week

At baseline (T0), patients choose one of the three exercise modules. The selected module had to be followed obligatory for the first six weeks. After 6 weeks (T1), the initial module could be replaced or amended by another study module. This or these selected module(s) had to be followed for another six weeks. The same procedure was repeated after 12 weeks (T2) of the exercise intervention. The main intervention ended after 12 weeks (T2). Thereafter, participants could voluntarily maintain one or up to three modules for another unsupervised 12-week time period. The follow-up ended at the 24-week time point (T3).

The supervised M1 took place once per week at a fixed, pre-scheduled time, via an online video conference platform. The training sessions lasted 60 min. They always started with a general 10 min warm-up, followed by a 40 to 45 min workout with specific exercises and finished with a 5 to 10 min stretching or relaxation part. The main workout focused on a different aspect of exercise each week (e.g., resistance training for lower extremities, sensorimotor training, home-based endurance training). The aim was for the YAs to learn exercises that they could do independently at home. For participants starting with M1, the study coordinator further recommended independent physical activity, such as walking or cycling, 1–2 times per week or to maintain the current volume of PA. Exercise recommendations for M2 were personalized and included primarily a combination of endurance and resistance training, 2–3 times a week, depending on the patient’s needs. Endurance training should be performed with moderate intensity for at least 30 min duration (or less, if the patient needed to adopt the exercise recommendations due to their current health status). The type of endurance exercise (e.g., walking, cycling, swimming) was chosen according to the individual interest of the patient. Resistance training consisted of various strength exercises for the large muscle groups (at least two each for lower and upper extremities and two for trunk muscles) and was aimed to improve muscular strength. Additional types of exercise (e.g., sensorimotor training) were added if therapy-related side effects were present. If necessary, exercise trainers conducted one introductory training session as a video conference to check for exercise techniques and answer any individual questions. Thereafter, M2 was executed as application-guided home-based intervention. M3 was executed in a certified exercise facility of the network OnkoAktiv and supervised by special qualified exercise trainers. The weekly recommendation was to participate in a personalized in-person exercise program 1–2 per week plus independent physical activity (or to maintain the current level of PA). Evolution of the training load was recommended in each of the three modules if this was possible for the patients.

Due to the differences in the three modules regarding frequency and content, the overall exercise recommendations in all study modules were guided by the present exercise guidelines of the ACSM, aiming to reach at least 150 min of moderate PA per week plus two strength-training sessions per week [16]. The subjects are asked to independently carry out that part of the overall recommendations that cannot be achieved via the module.

### 2.4. Outcomes and Study Instruments

#### 2.4.1. Physical Activity

Physical activity levels were determined by the standardized Godin–Shephard Leisure-Time Questionnaire [32]. The questionnaire was used to ask for pre-diagnosis-, post-diagnosis- and pre–post-intervention physical activity levels within three categories: light, moderate and vigorous physical activity in minutes per week. Participants were categorized in a sufficiently active and insufficiently active subgroup with a threshold of 150 min of moderate or 75 min of vigorous physical activity per week (or a combination of both) based on the ACSM guidelines.

#### 2.4.2. Module Selection and Exercise Preferences

The YOUEX study is based on a comprehensive evaluation questionnaire that focused on the main outcomes: module selection and exercise preferences. The individual module selections were queried and documented at the first three time points (T0, T1, T2). Reasons for any module selection and why other modules were not selected was collected through open answer questions while multiple answers were possible. The subgroups of the initial module selection were analyzed according treatment and employment status. Further, we asked for module preferences under COVID-19-free conditions.

#### 2.4.3. Impact of the COVID-19 Pandemic

The questionnaire about the impact of COVID-19 was self-developed and used internally in other studies at the National Center of Tumor Diseases (NCT), Heidelberg, but has not been published. The COVID-19 questionnaire consisted of six items and surveyed the impact of COVID-19 on a patient’s current job situation, leisure-time activities, physical activity levels, self-efficacy, anxiety and mental health. Further, we asked whether COVID-19 had any effect on the participant’s module selection. The COVID-19 questionnaire was submitted later during the ongoing study due to the COVID-19 lockdown in November 2020. Therefore, not all participants completed the COVID-19 questionnaire.

### 2.5. Statistical Analysis

The statistical analysis followed an exploratory approach applying descriptive and inferential statistics using the programs IBM SPSS Statistics 28 and Microsoft Excel 2016. The inference statistical pre-analysis for the normal distribution hypothesis was conducted using Shapiro–Wilk test and optical representation by histograms and Q-Q-diagrams. For inference statistics, non-parametric Wilcoxon, Friedman and Pearson chi-square tests were applied. We also conducted the Dunn–Bonferroni test as an equivalent post hoc procedure to the Friedman test. Correlations between the categorical variables were estimated applying Cramer’s V. A 95% confidence interval was defined for all significance tests and all tests were two-sided. Due to the exploratory approach, procedures for multiple test adjustments were dispensed [33]. Effect sizes for median differences were calculated using the Pearson correlation coefficient r. To measure the effect size of the Friedman test, we used Kandell’s W. Cramer’s V, Pearson’s r and Kandell’s W were reported according to the interpretation by Cohen (small ≥ 0.1; medium ≥ 0.3; large ≥ 0.5) [34].

### 2.6. Qualitative Analysis

The qualitative data were analyzed by structured content analysis in Microsoft Excel 2016. We coded all open answers and sorted them into categories based on Kuckartz et al. [35]. Then, we counted the number of codes (quantitative) and sorted them according to their number of occurrences.

## 3. Results

**Figure 1:**
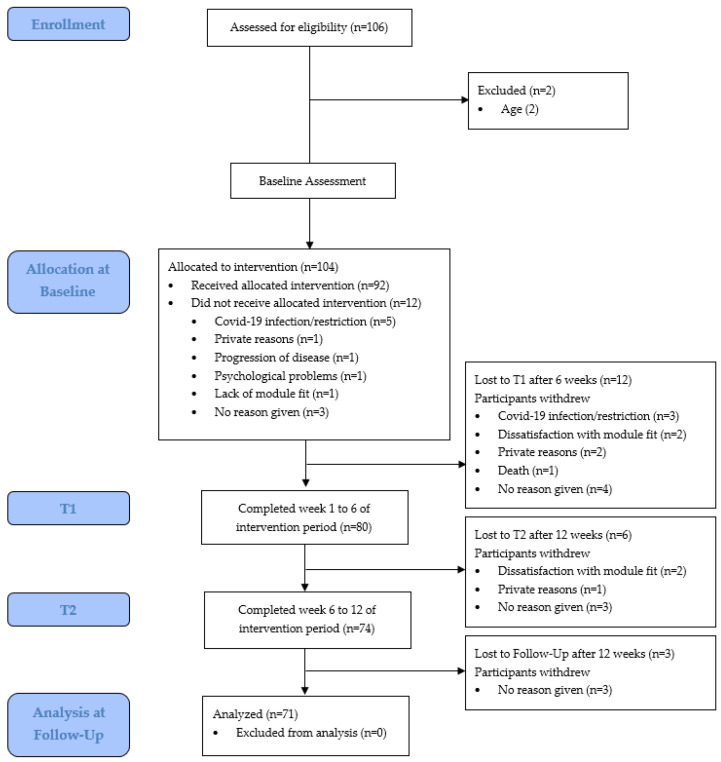
Flow diagram from enrollment to analysis, including dropouts.

From September 2020 to April 2021, 106 young adults contacted us regarding participation in YOUEX across all social media platforms which were deployed for recruitment. One-hundred and four of those met the inclusion criteria and 92 patients confirmed the letter of consent and started at baseline (T0). Figure 1 shows the flow diagram from enrollment to analysis including the number of and reasons for dropouts.

### 3.1. Patient Characteristics

Ninety-four percent of participants (*n* = 86) were women and the average age among all participants was 32 years (min: 19; max: 39). The distribution of cancer types was 55% breast, 19% (non-)Hodgkin lymphoma and 15% other cancer types (e.g., ovarian, skin, colon, cervix, testicles, lung). Overall, 18 YAs (20%) underwent acute tumor therapy (chemotherapy or radiation) at baseline. There were no smokers among the participants (see Table 1).

### 3.2. Physical Activity

During the primary intervention period the mean amount of light physical activity did not differ significantly between the time points (see Table 2). Significant improvements were found in both, moderate PA level (chi-square (3) = 23.556, *p* < 0.001, *n* = 70, W = 0.11) and vigorous PA level (chi-square (3) = 18.995, *p* < 0.001, *n* = 69, W = 0.09) across the time points. There was also a significant improvement in the total duration of PA (chi-square (3) = 18.199, *p* < 0.001, *n* = 66, W = 0.09). Using the Dunn–Bonferroni test, a significant differences in the duration of PA between post-diagnosis and T1 (moderate PA: z = 0.821, *p* = 0.001, r = 0.10; total PA: z = 0.758, *p* < 0.005, r = 0.09) and between post-diagnosis and T2 (moderate PA: z = 0.750, *p* < 0.005, r = 0.09; vigorous PA: z = 0.696, *p* < 0.01, r = 0.08; total PA: z = 0.795, *p* < 0.005, r = 0.10) were computed. The proportion of patients belonging to the sufficiently active subgroup increased from post-diagnosis (40%) to T1 (53%) to T2 (59%).

Compared to pre-diagnosis, the following significant differences were determined: The PA with vigorous intensity decreased significantly from pre-diagnosis to post-diagnosis (z = 0.819, *p* = 0.001, r = 0.10) and the PA with moderate intensity increased significantly from pre-diagnosis to T1 (z = 0.621, *p* < 0.05, r = 0.10). There was no significant change from pre-diagnosis to T2. Before diagnosis, 63% fulfilled the international physical activity recommendations of ACSM. This proportion dropped to 40% after diagnosis.

### 3.3. Module Selection and Exercise Preferences

#### 3.3.1. Initial Module Selection at T0

With regard to module preferences at baseline (T0), 50 participants (54%) chose the online-training app (M2), 32 participants (35%) chose the supervised, group-based online exercise program (M1) and 10 participants (11%) chose the in-person exercise program (M3). However, due to the COVID-19 national lockdown and several restrictions on exercise facilities, M3 could not be served from November 2020 to March 2021. Participants who performed M3 at that time could switch to either M1 or M2. The most frequently mentioned reason for choosing M2 (56% of a total of 82 qualitative answers) was the flexibility in terms of time. The second most given reasons were both, personal reasons (e.g., “I want to lose weight”) and the personalized training schedule (respectively 15% of 82 qualitative answers).The reasons for choosing M1 (a total of 56 qualitative answers were given) were the fixed training date (30%) and doing sports with other cancer patients (27%). Sixteen percent of the YAs indicated that M1 was easy to integrate into everyday life. The most common reason for choosing M3 (a total of 19 qualitative answers were given) was the individual supervision by an exercise therapist (47%). Additionally, fixed dates (26%), closeness to residence (16%) and social contact (11%) were mentioned in regard to M3.

#### 3.3.2. Module Change at T1

**Figure 2:**
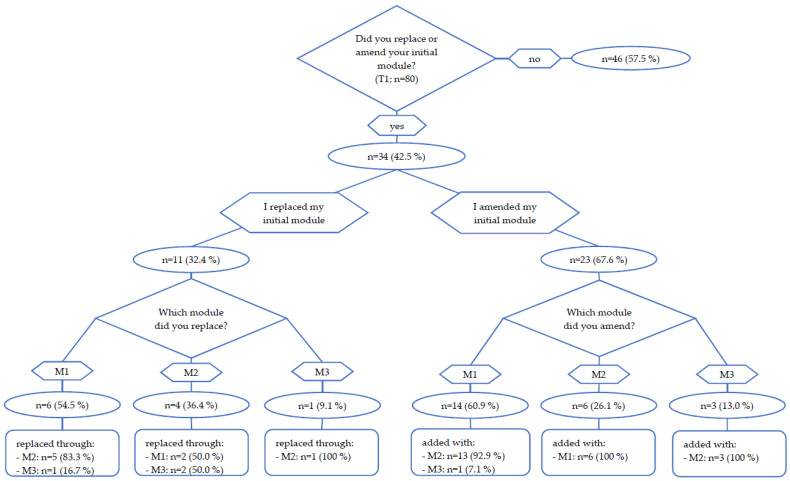
Module change at T1.

Eighty from ninety-two participants completed the first 6 weeks of intervention and reached the first time point of intervention (T1). At T1, 43% (*n* = 34) of participants replaced or amended their initial module (see Figure 2). Eleven participants (14%) replaced their initial module, of which 55% (*n* = 6) chose M2, 27% (*n* = 3) chose M3 and 18% (*n* = 2) chose M1. Twenty-three participants (29%) amended the initial module of which thirteen patients added M2 to M1 (57%), six patients added M1 to M2 (26%), three patients added M2 to M3 (13%) and one patient added M3 to M1 (4%). Reasons for replacing or amending the group-based online exercise program (M1) were the wish to increase activity through adding another module (33% of 39 given answers) and the wish to receive more individual advice by an exercise therapist (26% of 39 given answers). YAs who chose the online-training-app (M2) (a total of 44 qualitative answers were given) named the wish for more interaction with trainers (27%), problems with COVID-19 restrictions (23%) and that they wanted to try another module (14%) as reasons for replacing or amending the initial module. The most common reason for replacing M3 (a total of 7 qualitative answers given) was COVID-19 restrictions (43%). Seventy-four from eighty participants reached the T2 (12 week) time point and the end of the main exercise intervention. Between T1 and T2, 71% took part in one module and 29% took part in two different modules.

#### 3.3.3. Subgroup Analysis of Patient Characteristics According to Module Selection at T0

Table 3 shows the patients’ characteristics regarding treatment status and employment within the three exercise modules at T0. A total of 18 patients were undergoing acute therapy during their participation. These patients were distributed relatively evenly across the three modules (M1: 39%, M2: 33%, M3: 28%). Looking at distribution within the module selection, 50% of those who chose M3 were undergoing acute therapy during their participation, while only 22% from M1 and 12% from M2 were under ongoing therapy. The correlation analysis indicated a significant correlation between module selection and treatment status (chi-square (2) = 7.81, *p* = 0.02, V = 0.29). Of the 36 patients who were employed at the time of the intervention, the majority chose M2 (53%), 39% chose M1 and 8% chose M3. There was no significant correlation between module selection and employment status. Additionally, physical activity level, distress and social media behavior were analyzed but showed no significant correlations.

### 3.4. Impact of COVID-19 Pandemic

**Figure 3:**
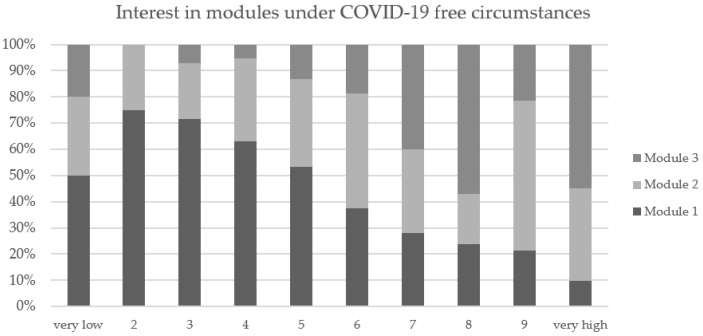
Interest in different modules under COVID-19-free circumstances on a scale of 1 (very low interest) to 10 (very high interest).

Eighty-five percent of the participants reported that the COVID-19 pandemic influenced their module selection. Thirty-three percent expressed that they felt unsafe to exercise in local facilities and were afraid of infection. More than half of all surveyed (52%) mentioned that their module selection was influenced by severe COVID-19 restrictions within the exercise institutions. The results coincide with the interest in different modules under COVID-19-free circumstances (see Table 4 and Figure 3).

The analysis of the general impact of COVID-19 illustrates that the status and circumstances of employment changed in 60% of the participants due to the COVID-19 pandemic (e.g., changing to home office (26 YAs), changing to short-time work (4 YAs) or other changes (16 YAs) such as constant new regulations as a teacher or extension of parental leave). The impact of the COVID-19 pandemic on the physical activity level was diverse across the YA population. While 39 YAs (53%) stated that their PA level had been reduced a little to a lot due to the COVID-19 pandemic, 27 YAs (29%) reported that they became more active during the pandemic. Seventy-five percent of all the participants felt, that their quality of life was impaired, 70% of the YAs felt stressed, 40% felt anxious and 37% felt helpless because of the COVID-19 restrictions during the pandemic.

## 4. Discussion

The YOUEX study investigated the exercise preferences and module selections of young adults with cancer. Our analysis revealed high acceptability and feasibility of online training programs. The YOUEX participants showed diverse preferences in exercise selection due to differences in cancer therapy status, interests in exercise options and favored level of supervision.

### 4.1. Physical Activity

Only 40% of all YOUEX participants met the physical activity recommendations after cancer diagnosis and therefore 60% can be classified as insufficiently active. This highlights the importance of exercise programming for YAs, especially right after their cancer diagnosis and during treatment. Interestingly, the participations in one or more of the three study modules had a significant effect on YAs moderate and vigorous PA levels after 12 weeks of the study intervention. Both activity levels increased by about 35%. The number of patients who met the ACSM guidelines after 12 weeks increased from 40% to 59%. A comparison of the self-reported physical activity level before and after cancer diagnosis revealed a significant difference in exercise intensity. YAs decreased their vigorous exercise by about 40% after diagnosis. This phenomenon has also been described by different authors in older cancer patients [37,38,39].

The effectiveness of exercise programs has been investigated by many researchers in the field of exercise oncology [4,11,12,40]. Friedenreich et al. [14] underlined the importance of post-diagnosis PA levels in their current review and meta-analysis. The authors revealed a significant difference in the mortality rates in cancer patients for those with low vs. high post-diagnosis PA levels. The benefits of physical activity for YAs have also been stated in the review by Munsi et al. [20]. Further, several European studies investigated the positive effects of exercise interventions in children and adolescents [28,41,42], pointing out that monitoring PA levels is important to accomplish positive PA effects [37].

### 4.2. Module Selection and Exercise Preferences

The evaluation of module selection at baseline showed the highest interest in module 2 followed by module 1. However, the strong influence of the COVID-19 pandemic and the comprehensive restrictions have had a high impact on module selections. Since module 3 could not be offered during the majority of the study period, no clear statement can be made about the actual interest in the in-person exercise program. Nevertheless, the hypothetical question about module interest under COVID-19-free circumstances showed very high interest in M3. At the same time, study participants showed high interest in both online modules (M1 and M2). After 12 weeks, more YAs participated in the individual home-based program M2 than the fixed group-based program M1. The most named reasons for choosing M2 was the flexibility in terms of time and individual training programming, whereas M1 was chosen because of the fixed training date and being motivated by others.

Further, the module changes at T1 highlighted interesting findings. Less than half of the participants changed their initial module. The amendment or replacement of modules were related to different reasons such as increasing the physical activity, interest in other modules or the wish to receive more individual advice by exercise trainers. Interestingly, despite the possibility to amend the initial module, no increase in PA could be determined between T1 and T2. Overall, comparing the two online modules, M1 (supervised, group-based online exercise program) was less popular than M2 (home-based individual training via app). Around 60% of those who chose M1 at baseline decided to amend or replace that module after 6 weeks. Compared to M2, only around 30% wanted to replace or amend that module. All in all, only around 20% of the YAs took part in two different modules during the 12-week intervention. Adams and colleagues outlined in their study with 533 AYA that the majority of patients preferred home-based (79%) and online (47%), but less hospital-based (25%) programs. Further, a significant higher proportion of AYA preferred individually supervised programs (82%) rather than group-based programs (63%). Interestingly, most AYA preferred to exercise ≥30 min on ≥3 days per week [31]. Another digital health intervention showed that a group-based intervention with a mobile app was accepted by YAs and revealed significantly greater improvements in muscle strength but had limited reach due to the competing needs experienced [43]. A systematic review on social media interventions targeting exercise in people with non-communicable diseases (including cancer) investigated five RCTs that improved the exercise behaviors and concluded overall feasibility of social media intervention among specific populations [44].

Our analysis highlights that exercise preferences of YAs are highly individual and diverse. First of all, the demography of patient characteristics showed the high variety of different diagnosis, treatment status, employment status, family situations and physical activity levels among YAs. At the same time, the given reasons for choosing a module or for not choosing a module were very diverse, some were even contradictory (e.g., flexibility in terms of time vs. fixed training dates). The subgroup analysis in which patient characteristics of the three different module groups were examined, brought only little insights. A significant correlation between treatment status and module selection was found. YAs undergoing acute therapy seem to prefer supervised training; however, due to the little sample size, non-randomization and limitations in the context of COVID-19 pandemic, we cannot conclude any clear statement about which exercise program fits the individual treatment status. Further, neither employment status nor physical activity level, distress or social media usage behavior seemed to have a definite influence on module selection. We therefore conclude that in order to be able to respond to different needs of YAs, a wide range of exercise programs must be created.

### 4.3. Implementation of the YOUEX Exercise Programs and the Impact of COVID-19

The comparatively small number of young cancer cases in Germany [45] leads to the challenge that region-specific group trainings might not be accessible to all patients. However, the need for high-quality exercise programs during and after cancer therapy still applies for YAs. Exercise programs offered digitally could be a suitable solution to consolidate YAs nationwide. Different studies analyzing digital health interventions showed the feasibility and acceptance in YAs with cancer [43,46,47]. Similarly, this study indicated that the digitally offered modules 1 and 2 were well-accepted. Especially during the COVID-19 pandemic, online-programs have had many advantages. Compared to the only in-person program (M3), major benefits of M1 and M2 were the independency against pandemic restrictions and the low risk of infections. However, M2 was intended as an unsupervised home-based program. Different studies show significant positive effects of the supervised training interventions on treatment-related side effects compared to unsupervised training [11]. Additionally, the qualitative evaluation of M1 and M2 revealed some critical aspects of online programs from the patient’s perspective. M1 was supervised in a group context but still a quarter of participants claimed that the training had not been individual enough. Still, online supervised training has its boundaries due to technical limitations (e.g., restricted field of vision). Further, one out of four patients in M2 (online, individual training plan) wished more personal interaction with the exercise therapist.

### 4.4. Limitations

Our study needs to be interpreted in light of several limitations. First, our data showed a great selection bias which resulted in a non-representable group of YAs. Our participants were mostly already active, with a high educational level, mostly women and non-smokers. We conclude that our reach was limited to the already interested, active group of potential YAs and people that were active on social media and engaged in self-help groups. In regard to the methodological approach, our study was a non-randomized intervention only, with no control group. A randomized, inactive control group could have shown causal differences in the patient-related outcomes and would have allowed a reasonable interpretation of our results. Further, when interpreting changes in the PA level it is imperative to consider that the study participants only subjectively estimated their PA level, there was no objectively measured method. Götte et al. highlighted that PA should be assessed by objective methods in pediatric cancer patients [48]. By using the Godin–Shepard Leisure-Time Questionnaire no distinction can be made between endurance or resistance training. Additionally, the study questionnaire did not cover the concrete PA levels at baseline and only asked for the pre- and post-diagnosis PA levels. In addition, there is a lack of data on the adherence of the participants to each module, which must be taken into account when interpreting the results. The different modules vary in terms of frequency, content and volume. A comparison of the modules with regard to the effects of each module on physical activity is therefore not possible. We also included a self-developed questionnaire about the impact of COVID-19, three months after we had started the patient recruitment. Overall, the YOUEX study was intensely impacted by the COVID-19 pandemic. The COVID-19 restrictions biased our measured outcomes (e.g., PA level), module selections and the entire execution of M3. The impact of COVID-19 on our study outcomes needs to be respected with important meaning.

### 4.5. Further Research

YOUEX has shown that social media tools are effective for participant recruitment in our young target group. However, it is not clear how to reach the broad range of YAs regarding their interest in PA participation, cancer diagnosis or treatment status. Further, the question of how to ensure adequate training stimuli for the right dose-effect in the context of online and/or home-based exercise programs remains unanswered and should be the subject of further research. Therefore, more data on the adherence of YAs participating in (online) exercise programs is needed. Additionally, the long-term effects of online exercise programs in YAs are yet to be evaluated and should be taken into account in future studies. Additionally, the question of how special exercise offers for YAs can be implemented into existing healthcare structures remains problematic. Further interventions should focus on the adaption, long-term implementation strategies and maintenance of exercise programs (including the long-term adherence of YAs) to provide a sustainable impact on PA levels and health-related improvements for this target group.

## 5. Conclusions

We found that young adults with cancer recruited via social media and different online websites have a wide range of interests and needs regarding exercise programs during and after cancer therapy. According to our findings, YAs need specific exercise programs that include their individual interests and needs. In this context, online exercise programs in different forms (e.g., group-based, individual program) can be an addition and/or an alternative to existing exercise options. It must be underlined, that such online programs were highly accepted in our study (which partly took place during lockdown periods in the COVID-19 pandemic) and can be effective in increasing YA’s physical activity levels.

## Figures and Tables

**Table 1:** Patient characteristics at baseline.

Characteristics	*n* (%)	Mean ± SD	Median	Range
Age (years)	92	31.9 ± 4.9	32.5	19–39
Gender				
Female	86 (93.5)			
Male	6 (6.5)			
Body composition				
Height (cm)	92	169.9 ± 7.1	170.0	146–190
Weight (kg)	92	68.2 ± 11.5	66.5	48–102
BMI (kg/m^2^)	92	23.6 ± 3.8	23.3	17.5–35.7
Disease				
Breast cancer	51 (55.4)			
(Non-)Hodgkin lymphoma	17 (18.5)			
Sarcoma	4 (4.3)			
Leukemia	3 (3.3)			
Brain tumor	3 (3.3)			
Other	14 (15.2)			
Disease progression				
Metastasis	17 (18.5)			
Relapse	13 (14.1)			
Treatment				
Surgery	68 (73.9)			
Chemotherapy				
Total	84 (91.3)			
Ongoing	18 (19.6)			
Radiotherapy				
Total	49 (53.3)			
Ongoing	1 (1.1)			
Immunotherapy				
Total	10 (10.9)			
Ongoing	5 (5.4)			
Hormone therapy				
Total	35 (38.0)			
Ongoing	26 (28.3)			
Other therapy				
Total	22 (23.9)			
Antibody	15 (16.3)			
Stem cell transplantation	5 (5.4)			
Other	2 (2.2)			
Education				
Middle school	2 (2.2)			
Vocational training	9 (9.8)			
University entrance qualification	24 (26.1)			
University degree	54 (58.7)			
Other degree	3 (3.3)			
Employment				
Employed, currently working	36 (39.1)			
Employed but on medical leave	35 (38.0)			
Still in education	17 (18.5)			
Housewife/houseman	1 (1.1)			
Retired	2 (2.2)			
Unemployed	1 (1.1)			
Family situation				
Married/permanent relationship	57 (62.0)			
Single	32 (34.8)			
Divorced	2 (2.2)			
Smoking behavior				
Smoker	0 (0)			
Non-smoker	92 (100)			
Social media usage behavior				
Smartphone/tablet (h/week)	90	12.5 ± 8.5	10.7	1–42
PC (h/week)	91	11.8 ± 15.1	4	0–60
Frequency of social media use (h/week)				
Instagram	91	3.4 ± 1.0	4	1–4
Facebook	91	2.6 ± 1.2	3	1–4
YouTube	91	2.3 ± 0.8	2	1–4
Twitter	91	1.2 ± 0.5	1	1–4
Tik Tok	91	1.1 ± 0.4	1	1–4
Twitch	91	1.1 ± 0.3	1	1–4
NCCN Distress ^1^	91	6.18 ± 2.1	6	1–10

^1^ National Comprehensive Cancer Network (NCCN) Distress thermometer: scale 0 (not stressed at all) to 10 (extremely stressed) [36].

**Table 2:** Physical activity before and during YOUEX intervention.

	Pre-Diagnosis		Post-Diagnosis		T1		T2		*p* Value
	*n*	M ± SD	*n*	M ± SD	*n*	M ± SD	*n*	M ± SD	
Light PA (min/week)	69	154.8 ± 142.7	69	194.1 ± 176.2	69	205.9 ± 203.1	69	206.2 ± 236.4	0.270
Moderate PA (min/week)	70	83.4 ± 81.4	70	76.1 ± 95.4	70	119.4 ± 116.2 *^,#^	70	116.8 ± 94.8 *	<0.001
Vigorous PA (min/week)	69	86.1 ± 94.5	69	46.9 ± 71.7 ^#^	69	68.3 ± 77.5	69	69.4 ± 65.6 *	<0.001
Total PA (min/week)	66	325.8 ± 210.9	66	322.0 ± 245.7	66	397.1 ± 256.3 *	66	399.9 ± 315.7 *	<0.001

^#^ significantly different to pre-diagnosis; * significantly different to post-diagnosis.

**Table 3:** Patient characteristics according to module selection at T0.

	M1 (*n* = 32)		M2 (*n* = 50)		M3 (*n* = 10)	
	*n*	%	*n*	%	*n*	%
Treatment status *						
During acute therapy	7	21.9	6	12.0	5	50.0
Before or after acute therapy	25	78.1	44	88.0	5	50.0
Employment						
Employed	14	43.8	19	38.0	3	30.0
On medical leave	14	43.8	17	34.0	4	40.0
Still in education	4	12.5	12	24.0	1	10.0
Not employed	0	0.0	2	4.0	2	20.0

* significant correlation with module selection.

**Table 4:** Interest in different modules under COVID-19-free circumstances on a scale of 1 (very low interest) to 10 (very high interest).

	*n* (%)	Mean ± SD	Median	Range	Percentile	
					25	75
M1	68 (73.9)	5.1 ± 2.6	5.0	1 to 10	3.0	7.0
M2	68 (73.9)	7.2 ± 2.8	7.5	1 to 10	5.0	9.5
M3	68 (73.9)	8.4 ± 2.1	9.5	1 to 10	7.0	10.0

## Data Availability

The datasets used and analyzed during the current study are available from the corresponding author upon reasonable request.
